# P269: Infection control practices during labor and delivery and newborn care in resource limited settings: assessment and recommendations for improvement

**DOI:** 10.1186/2047-2994-2-S1-P269

**Published:** 2013-06-20

**Authors:** WC Huskins, V Manchanda, N Singh

**Affiliations:** 1 Division of Pediatric Infectious Diseases, Mayo Clinic, Rochester, MN, USA; 2 Clinical Microbiology and Infectious Diseases, Chacha Nehru Bal Chikitsalaya, New Delhi, India; 3 Division Infectious Diseases, Children's National Medical Center, Washington, DC, USA

## Introduction

India is amongst the countries with high maternal mortality rate (MMR) & infant mortality rate (IMR). Most of these deaths occur due to sepsis. UNICEF initiated a review of infection control practices (ICP) during labor & delivery (L&D) & in newborn care at two states of Rajasthan & Odisha, India.

## Objectives

To assess current ICP during L&D and newborn care with an aim to improve ICP to reduce MMR & IMR due to infections.

## Methods

A two member consultant team was constituted by UNICEF Delhi, India. The team conducted a structured assessment of different facilities (sub-district and district hospitals in Rajasthan & Odisha) on ICP in L&D rooms & Special Care Newborn Units (SCNUs). The team completed the assessments using the Infection Control Assessment Tool [1,2]. Draft reports were provided to the respective offices on the evening of the assessments.

## Results

Assessments were completed in 5 community health centers and district hospitals with L&D rooms and SCNUs. Defined systems for cleanliness and general hygiene were conspicuously absent. Hand hygiene practices were poor due to lack of awareness & supplies. Biological waste segregation was not appropriate and there were concerns regarding storage and disposal of bio-medical waste. Exposure risk to healthcare workers including doctors, nursing staff, and support staff is area of major concern.

## Conclusion

The National Program has provided funds and brought enormous patient load to healthcare setting. However, training and uninterrupted supplies for L&D and SCNU must be ensured. Specific recommended role of UNICEF and state health ministry of two states were highlighted.

## Competing interests

None declared
